# Ultra-wide-band structural slow light

**DOI:** 10.1038/s41598-018-33090-x

**Published:** 2018-10-04

**Authors:** Yiming Lai, Mohamed Sabry Mohamed, Boshen Gao, Momchil Minkov, Robert W. Boyd, Vincenzo Savona, Romuald Houdré, Antonio Badolato

**Affiliations:** 1 Department of Physics University of Ottawa, Ottawa, Ontario K1N 6N5 Canada; 2 Center for Nanoscale Science and Technology, National Institute of Standards and Technology, Gaithersburg, MD 20899 USA; 3 Institut de Physique, Ecole Polytechnique Fédérale de Lausanne (EPFL), CH-1015 Lausanne, Switzerland; 4 The Institute of Optics, University of Rochester, Rochester, NY 14627 USA

## Abstract

The ability of using integrated photonics to scale multiple optical components on a single monolithic chip offers key advantages to create miniature light-controlling chips. Numerous scaled optical components have been already demonstrated. However, present integrated photonic circuits are still rudimentary compared to the complexity of today’s electronic circuits. Slow light propagation in nanostructured materials is a key component for realizing chip-integrated photonic devices controlling the relative phase of light and enhancing optical nonlinearities. We present an experimental record high group-index-bandwidth product (GBP) of 0.47 over a 17.7 nm bandwidth in genetically optimized coupled-cavity-waveguides (CCWs) formed by L3 photonic crystal cavities. Our structures were realized in silicon-on-insulator slabs integrating up to 800 coupled cavities, and characterized by transmission, Fourier-space imaging of mode dispersion, and Mach-Zehnder interferometry.

## Introduction

The engineering of frequency dispersion in light-guiding photonic crystal (PC) structures is one of the most promising research avenues in the field of slow-light^1–4^. The primary goal of such device-research is to achieve structural slow-light operation over the largest possible bandwidth, with large group index, minimal index dispersion, and constant transmission spectrum. Such features are required to enable multimode or pulsed operation as they suppress pulse distortion and the onset of echoes. A commonly adopted figure of merit for this set of features is the group-index-bandwidth product (GBP). Significant progress in recent years has led to the creation of photonic structures with increasingly high GBP values^5^. Here, we report on the experimental demonstration of a record high GBP in silicon-based coupled-cavity waveguides (CCWs)^6–11^ operating at telecom wavelengths.

## Results and Discussion

**Figure 1:**
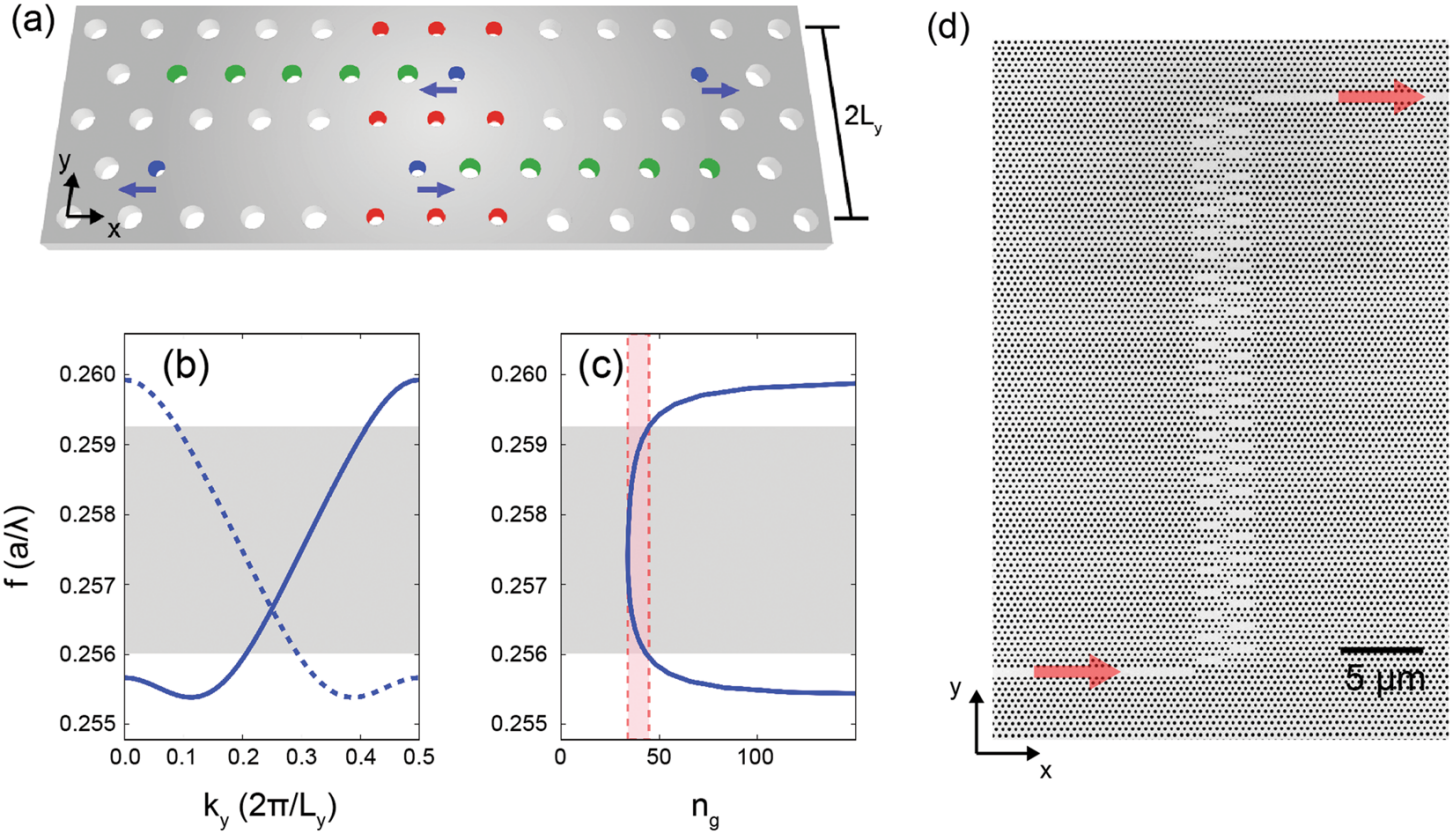
(**a**) Schematic of the CCW unit cell. The radii of the colored holes are modified (Δ*r*_1,2,3_) with respect to the PC bulk holes. The blue holes are also shifted outward (Δ*x*). (**b**) GME-simulated band structure and (**c**) corresponding *n*_*g*_ in normalized frequency units (*a*/*λ* = *ωa*/2π*c*). The solid line corresponds to the guided mode in a representation where the period of the structure is assumed to be L_y_. The dashed line indicates the folded band, in an equivalent representation where instead the period is taken as the elementary cell, of length 2L_y_, containing the two staggered cavities. The operational bandwidth Δf is marked by the gray region [in panels (b and c)]. The pink region in (**c**) indicates the region where *n*_*g*_ deviates from a mean value (〈*n*_*g*_〉 (by less than ±10%. (**d**) SEM top view image of a 50-CCW. Red arrows indicate the light input and output.

Our results rely on novel CCW designs, optimized using a genetic algorithm, and refined nanofabrication processes^12^. The schematic design of our CCW unit cell is shown in Fig. 1a. It comprises two L3 photonic crystal cavities^13^ (PCCs) separated by 5*a* in the *x*-direction and *a*√3 in the propagation direction *y* (where *a* is the lattice constant of the PC). Defining the CCW flat-band operation bandwidth as the spectral range Δ*ω* where the group index (*n*_g_ = c d*k*/d*ω*) deviates from a mean value 〈*n*_g_〉 by less than^5^ ±10%, we maximized the group-index bandwidth product (GBP) (=〈*n*_g_〉 Δ*ω*/*ω* for *n*_g_ ~ 〈*n*_g_〉), while concomitantly minimizing losses, through the combination of three criteria. First, the shortest possible spatial period results in a large Brillouin zone and thus in a large GBP for a given bandwidth. Second, the first-neighbor coupling must be kept small, in order to minimize the influence of higher-order cavity modes, so that the guided band mainly arises from the coupling between the fundamental modes of each single PCC. To achieve this condition, we adopted the staggered geometry, where the mode overlap between first neighbors is naturally reduced. Third, the maximization of the quality factor (*Q*) of each single L3 PCCs^14^ does not play any role in determining the GBP, however, it minimizes the intrinsic losses of the CCW. Our PCCs, which are all strongly coupled, are expected to have a *Q* between the unmodified L3 PCC (~7700) and the singly optimized L3 PCC (~220,000) achieved through the modification of Δ*r*_3_ and Δ*x*. We carried out the optimization procedure via a genetic algorithm^15,16^ combined with the guided mode expansion (GME) method^17^. As an objective function, we choose the GBP with an additional price if the maximum radiation loss per unit time (*L*_e_) of the electric field intensity exceeded *L*_e_ = 100 dB/ns. To maximize the objective function, we introduced four free parameters (Fig. 1a, Δ*r*_1,2,3_ and Δ*x*) that were varied simultaneously. Radii and positions of the blue air holes (Δ*r*_3_ and Δ*x* respectively) mostly affected the *Q* of each L3 PCCs. Radii of red and green air holes (Δ*r*_1_ and Δ*r*_2_ respectively) instead affected primarily the first- and second-neighbor couplings respectively. Figure 1b,c show the computed band structure of a genetically optimized CCW and the corresponding n_g_. (The folded band representation, indicated by the dashed line, helps to model forward and backward propagating modes measured via Fourier space imaging, as showed hereafter.) Our numerical simulations demonstrated a GBP value of 0.47, over an 18.0 nm bandwidth, for the set of parameters (Δ*r*_1_, Δ*r*_2_, Δ*r*_3_, Δ*x*) = (−0.0385a, −0.0279a, −0.0759a, 0.1642a). Higher theoretical GPB values of up to 0.66 were obtained at the expense of a narrower bandwidth (see Supplementary Section S3). To achieve CCW performance with a flat-band centered near 1550 nm and with standard silicon membrane thickness of *d* = 220 nm (see Methods), we locked the lattice constant *a* = 400 nm and bulk air hole radius *r* = 0.25*a*.

Figure 1d shows a top view scanning electron microscope image of one of our CCWs composed of 50 PCCs (50-CCW) fabricated in silicon-on-insulator (see Methods). As detailed in the Supplementary Section S1, all the CCWs were coupled to two separate input-output PC waveguides (red arrows in Fig. 1d) each of which (not shown in the figure) was butt-coupled to a silicon-strip waveguides and then to a spot size converter for efficient end-fire coupling. A nano-tether-based structure surrounding the device (not shown in figure) was engineered to achieve buckling-free suspended membranes^18^.

**Figure 2:**
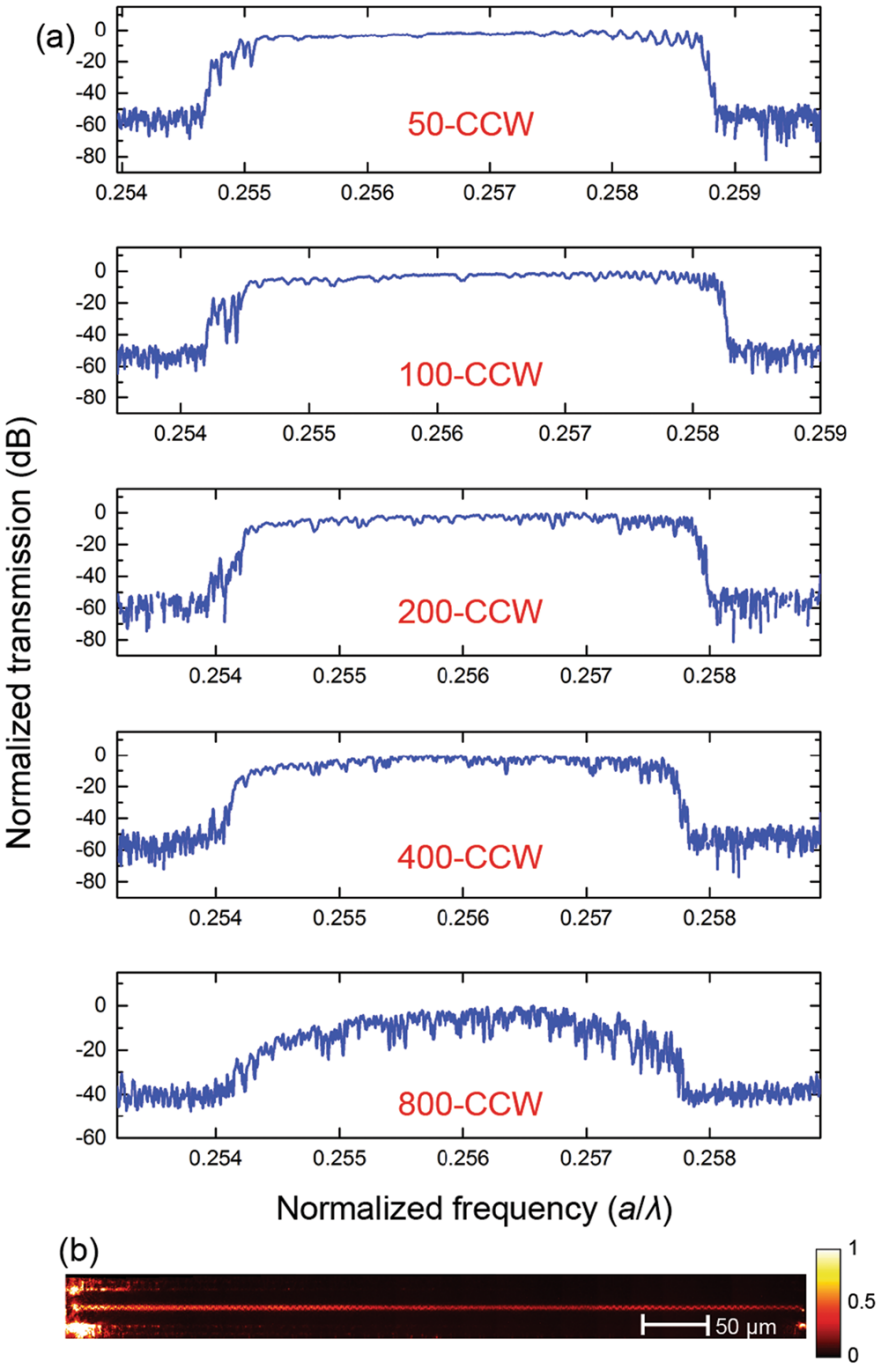
(**a**) Normalized transmission spectrum of CCWs made up of 50, 100, 200, 400, and 800 PCCs, respectively. (**b**) Optical image of an 800-CCW when light is propagating from left to right.

To study the transmission of the designed CCWs with respect to the number of PCCs composing the CCWs, we fabricated five groups of CCWs formed by 50, 100, 200, 400, and 800 total PCCs. Figure 2a shows the typical transmission profiles of those groups over their operation bandwidth. The transmission remained flat with variation <10 dB over a wide-band for CCWs containing up to 400 PCCs (400-CCW). For the 800-CCW, the transmission started dropping as extrinsic optical scattering losses (likely due to structural disorder and material absorption) became significant^19^, as also hinted by the more pronounced transmission fluctuations^20^. In general, for all CCW groups, part of the transmission fluctuations, especially at the extrema of the slow light band, display regularities and are thus likely caused by the abrupt coupling of the CCW to the input and output waveguides. A proper apodization of the design^5^ may prevent this effect and further improve the performance of the device. Figure 2b shows the real-space image of an 800-CCW as light propagates along the waveguide. The propagation loss of the CCW was estimated first by filtering out the strong scattering at the input edge of the CCW and then by integrating the real space profile along the *x*-direction. From the resulting intensity versus *y*-position, we fitted the loss per unit length^21^. By combining it with the measured *n*_g_ (discussed later), the radiation loss per unit time at the center of the transmission band (1562.5 nm) was determined to be *L*_e_ ~ 56 dB/ns (~76 dB/cm).

To comprehensively investigate our different CCWs we applied two complementary techniques: Fourier-space imaging (FSI)^22^ and Mach-Zehnder (MZ) interferometry^23^.

**Figure 3:**
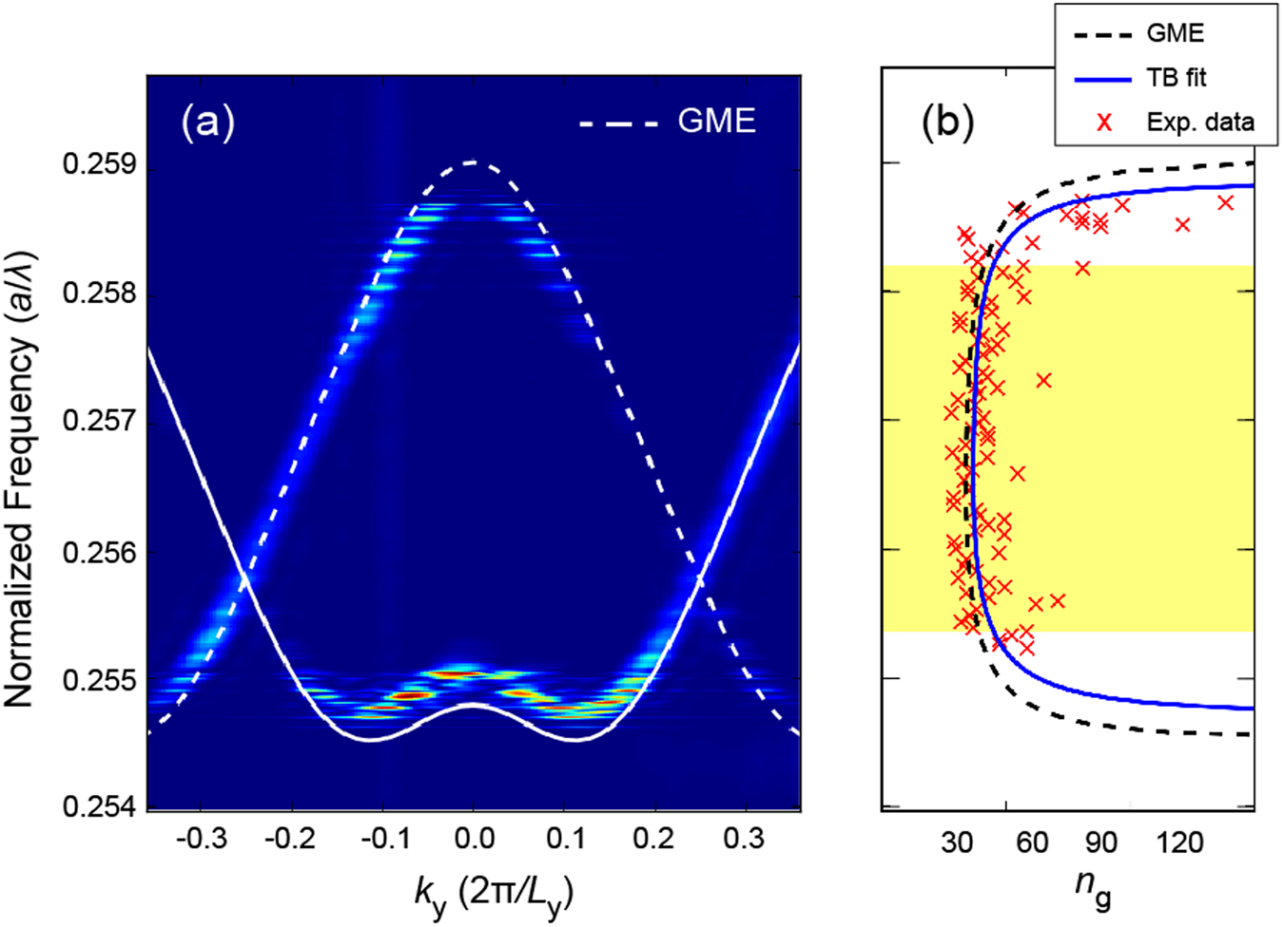
(**a**) Photonic band structure of a CCW made up of 50 PCC_S_ (50-CCW) measured by FSI overlaid with the GME simulation (white dashed line). (**b**) Red crosses: experimental *n*_*g*_ calculated from data in (**a**). Blue continuous curve: fit of the experimental *n*_*g*_ using the TB model. Black dashed curve: prediction of the GME simulation.

For FSI, the out-of-plane emission of the coupled PCCs is linked to the wave vector of the propagating mode through momentum conservation at the silicon/air interface. By measuring the far-field radiation angle, the in-plane Bloch wave vector *k*_y_ of the mode is determined, and the dispersion relation can be reconstructed by scanning the light frequency. Figure 3a shows the measured dispersion relation for a 50-CCW and the excellent agreement with the simulated dispersion relation using the GME method (dashed white curve). From the measured dispersion relation, we obtained by numerical differentiation of the peak intensities the *n*_g_ of the CCW (red crosses in Fig. 3b), which are compared with the *n*_g_ curves obtained from the tight-binding (TB) model (blue curve) and the GME method (dashed black curve). By fitting the experimental data with the TB model, we determined 〈*n*_g_〉 = 41.0 and Δ*λ* = 17.7 ± 0.5 nm, corresponding to GBP = 0.47 ± 0.01 (with the error accounting for the uncertainty in fitting the dispersion curves). The prediction of the GME model for the same device gave 〈*n*_g_〉 = 37 over an operational bandwidth of Δ*λ* = 18.0 nm. To our knowledge, this is the highest experimental GBP ever reported in PC-based slow light devices. It is also worth noting the excellent agreement between experimental data and theory, with the measured GBP being nearly the same in all fabricated devices of nominally the same design (with the highest GBP observed in the 200-CCW). To explore the space of parameters around this optimal design we fabricated three additional series of CCW devices. For these devices, we measured a higher group index, at the expense of consistently lower GBP values, as detailed in the Supplementary Section S3.

**Figure 4:**
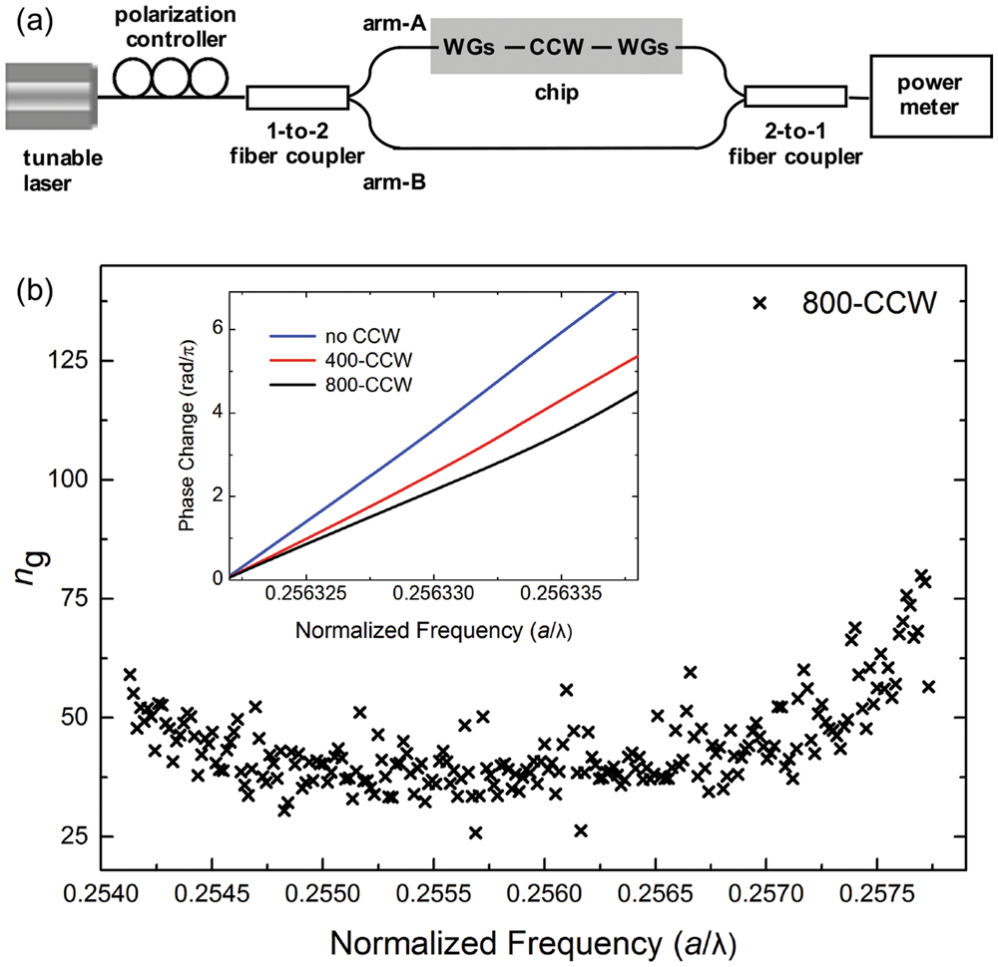
(**a**) Schematic of the optical fiber-based Mach-Zehnder interferometer set up. Each CCW was singly coupled to a left and right on-chip waveguide system (WG). (**b**) Calculated *n*_*g*_ when a 800-CCW was present in arm-A. (Inset) Change in the relative phase, Δ*φ*(*ω*) − Δ*φ*(*ω*_0_), with respect to *ω*_0_ = 2π*c*/(1560.54 nm), as obtained from the MZ fringes when in arm-A there was: no CCW (blue), a 400-CCW (red), and a 800-CCW (black). The slope of the curves decreased when longer CCWs were inserted as the arm length difference decreased.

To confirm the FSI results, we carried out an alternative experimental investigation based on a MZ interferometer^23^ (Fig. 4a) consisting of a CCW in one arm (arm-A) and an external optical fiber in the second arm (arm-B). (See also Supplementary Section S4) Fig. 4b (inset) shows the change in the relative phase for three configurations of the MZ. Figure 4b shows the calculated group indices when an 800-CCW was inserted in the arm-A. The results are in excellent agreement with the *n*_g_ measured via FSI. The two techniques are complementary, with the MZ method more suited for measuring longer CCWs (given that the uncertainty in n_g_ scales with 1/*L*_c_) and the FSI more suited to local investigations of the operating structure.

Together with setting a new record in the GBP of PCC-based CCWs, the nanophotonic structures reported in this letter have many potential applications as building blocks in on-chip slow-light-based devices. Particularly attractive are the implementations of slow-light in signal processing^24^ and slow-light-enhanced spectroscopic interferometers^25^. In the latter, the resolution can be increased by a factor as large as *n*_g_ and, as demonstrated here, this performance can be extended over a ultra-broad bandwidth. High resolution spectrometers integrated in chip-scale platforms can find transformative applications in chemical and biosensing.

## Materials and Methods

The silicon-on-insulator wafer consisted of a 220 nm top silicon layer and a 3 μm buffer oxide layer on a silicon substrate. The CCW pattern was defined by 100 kV electron beam lithography direct writing using a positive e-beam resist. The pattern was transferred from the resist (ZEP-520A) to the silicon top-layer by fluorine based inductively coupled plasma dry etching. To undercut the buried oxide layer, we used wet buffered oxide etchant while protecting the spot size converter by photo-resist. Engineered lateral openings in the membrane made the suspended structures free from buckling.

## Electronic supplementary material

Supplementary Information
