# Supplementary material for: Ultra-wide-band structural slow light
Source: Sci Rep. 2018 Oct 4;8:14811. doi: 10.1038/s41598-018-33090-x (PMC6172281; doi:10.1038/s41598-018-33090-x)
Supplement: Supplementary file 1 — Supplementary Information [file 41598_2018_33090_MOESM1_ESM.docx]

**Ultra-wide-band structural slow light**

Yiming Lai^1,2^, Mohamed Sabry Mohamed^3^, Boshen Gao^4^, Momchil Minkov^3^, Robert W. Boyd^1^, Vincenzo Savona^3^, Romuald Houdré^3^, and Antonio Badolato^1,2*^

*^1^Department of Physics University of Ottawa, Ottawa, Ontario K1N 6N5, Canada*

*^2^Center for Nanoscale Science and Technology, National Institute of Standards and Technology, Gaithersburg, MD 20899, U.S.A.*

*^3^Institut de Physique, Ecole Polytechnique Fédérale de Lausanne (EPFL), CH-1015 Lausanne, Switzerland*

*^4^The Institute of Optics, University of Rochester, Rochester, NY 14627, U.S.A.*

**Supplementary Information**

**S1. CCW input-output coupling**

An end-fire design was implemented to couple light into and out of our CCW^[[1]](#endnote-1)^. A continuous wave (CW) laser with tuning range from 1480 nm to 1660 nm was butt-coupled to the chip via a tapered and lensed optical fiber. A spot-size converter, comprising a SU-8 polymer waveguide with mode-field diameter matched to the lensed fiber, was used to collect the light from the lensed fiber (Fig. S1a). The coupled light was then adiabatically transferred from the SU-8 waveguide to the Si strip waveguide via an inverse taper waveguide. A strip waveguide-PC waveguide coupler was used to couple the light into the PC waveguide and the CCW (Fig. S1b). The transmission spectrum of the CCW was collected likewise by a different lensed fiber at the opposite facet.


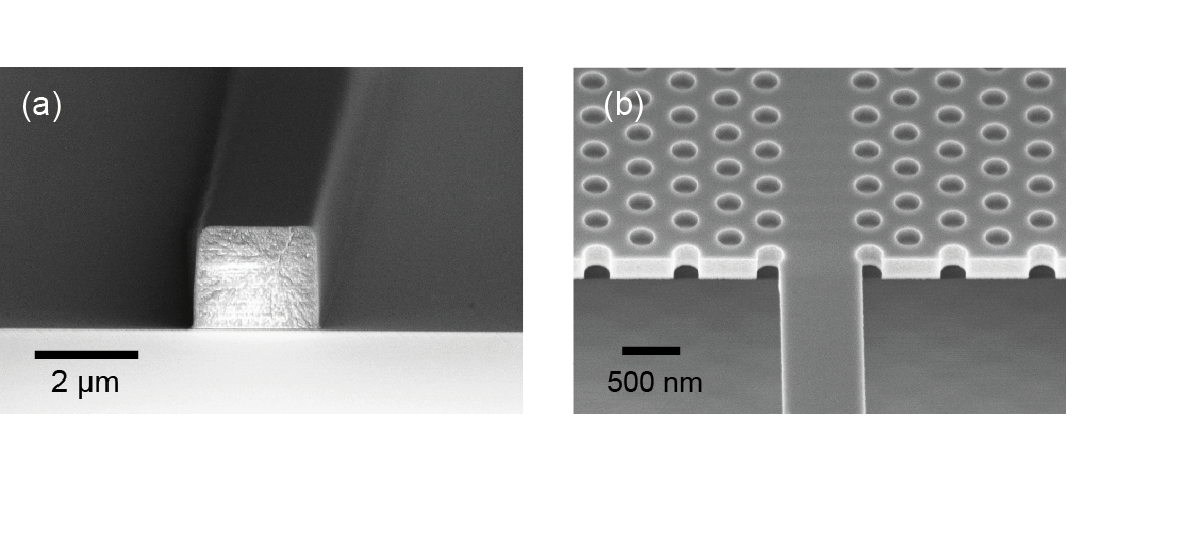


**Figure S1 |** Cross-section SEM images of the (a) SU8 waveguide and (b) the interface between the Si strip waveguide and the PC waveguide.

**S2. Fourier-space Imaging**

The Fourier-space Imaging **(**FSI)^[[2]](#endnote-2)^ was performed first by collecting the radiation of the CCW positioned at the focal plane of a high numerical aperture (NA) objective. A spatial filter was applied at the conjugate image plane to select the region of interest along the CCW and to suppress stray light, before transforming the collected light by a lens that focused the light onto a CCD detector to capture the Fourier plane. The experimental acquisition showed in Fig. 3 displayed the dispersion relation of both forward and backward propagating modes of the CCW, appearing mirrored about the symmetry axis (*k*_y_ = 0) and folded at the first Brillouin zone boundary (*k*_y_ = ± π/2*L*_y_). The measured intensity was in proportion to their respective radiative contribution, given that the CCW modes lie above the light line. The forward propagating mode was clearly dominating the transport channel, especially in the central constant *n*_g_ range of the bandwidth, where the CCW operated in the dispersive regime^[[3]](#endnote-3)^.

A quasi‑continuous dispersion relation could be traced through the experimental data, which was dictated by the collective spectral response of the constituent cavities. The inherent discretization of states due to the finite cavity number was apparent especially with the shorter CCWs, since the CCW design utilized a broad bandwidth span relative to the linewidth of the formed states. The dispersion profile smoothed when the CCW chain was elongated, with a proportional number of states being created, which narrowed down the inter‑state frequency gaps.

Moving towards the edge of the band, *n*_g_ gradually rose and scattering into the backward-propagating mode became progressively more significant as a consequence of disorder‑induced scattering. When *n*_g_ exceeded a value of around 50, the dispersion linewidth, which is naturally governed by the finite-sample size and attenuation, began to broaden, signaling the onset of diffusive light transport and subsequent localization in the photonic crystal lattice towards higher *n*_g_ values. Localized states exhibited spectral signatures with a broad extent in *k*‑space and corresponding dips in light transmission.

**S3. Additional series of CCW devices**

To explore the space of parameters around the GBP optimal design reported in the article (here termed D1) three additional CCW designs targeting higher *n*_g_ were realized using the same fabrication protocols and nano-tether-based structure. Table S1 compares the experimental results for all four designs. D1 is the design described in the article, which exhibits the highest GBP. Fig S2 shows an example of the measured FSI and normalized transmission for a D4 design with a CCW formed by 50 coupled PCCs. Our measurements showed systematically lower GBPs and lower transmission bandwidths for D2-4. This was an expected result because a higher *n*_g_ implies a higher susceptibility to fabrication imperfections. We emphasize, however, that D2 represents still one of the PCN-based CCW slow light devices with largest bandwidth at such high *n*_g_.

|  | 〈*n*_g_〉 | Δ*λ* | GBP |
| --- | --- | --- | --- |
| D4 | 47.0 | 12.4 | 0.38 |
| D3 | 81.2 | 5.8 | 0.30 |
| D2 | 94.5 | 3.1 | 0.19 |
| D1 | 41.0 | 17.7 | 0.47 |

**Table S1** | Average index of refraction (〈*n*_g_〉) and bandwidth (Δ*λ*) measured for three additional series of CCW devices. D1 corresponds to the device reported in the article.

**
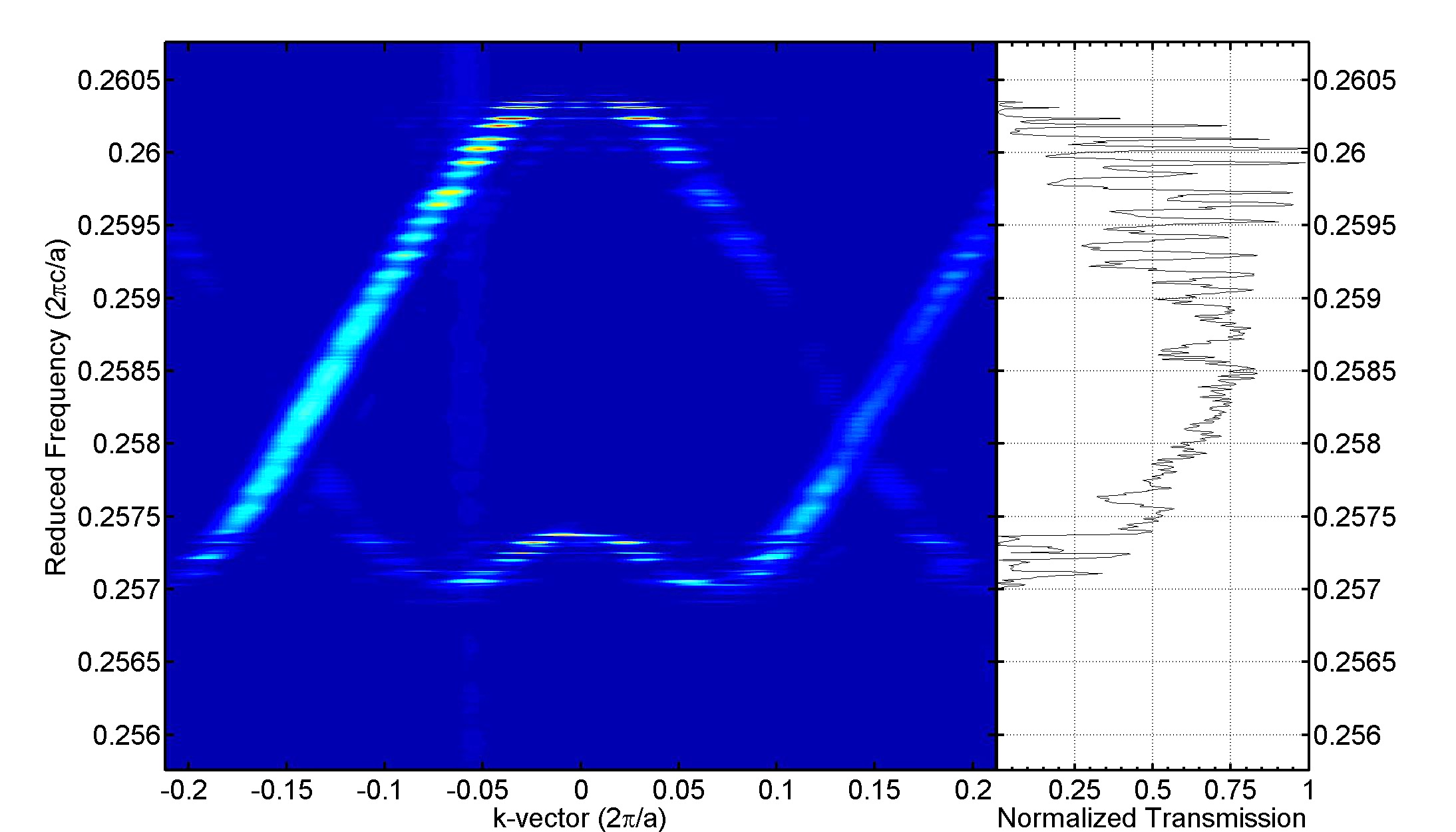
**

**Figure S2** **|** (a) Photonic band structure measured by FSI and normalized transmission of a D4 design CCWs formed by 50 PCC_S_ (50-CCW).

**S4. CCW-based Mach-Zehnder interferometry**

Interference in our CCW-based Mach-Zehnder (MZ) (Fig. 4a) was measured as a function of the input laser wavelength. When in arm-A the two tapered fibers were directly coupled through a small air-gap (i.e., the chip was entirely removed), the MZ fringes (Fig. S3 inset) showed the optical path in the arm-B to be ~ 6 cm longer than in the arm-A. We defined the relative phase between the two arms without chip as *φ*_r0_ = *φ*_B_ – *φ*_A_ and with the chip as *φ*_r_ = *φ*_r0_ – *ω (n*_c_*L*_c_ *+ n*_w_*L*_w_*)/c,* where we defined a CCW with effective index *n*_c_ and length *L*_c_, and the chip-integrated input-output coupling waveguides (indicated as WGs in the schematics of Fig. S3a) with effective index *n*_w_ and total length *L*_w_. As we scanned the laser frequency, the change in the relative phase

was *dφ*_r_ /*dω* = *dφ*_r0_ /*dω* – *(n*_g_ *L*_c_ *+ n*_gw_ *L*_w_*)/c,* where *n*_g_ and *n*_gw_ are the group indices of the CCW and the coupling waveguides. From the chip design and the MZ fringes observed in presence of the different CCWs, we calculated that the entire left and right couplings waveguide system contributed for about 1.2 cm to the optical path length of the arm-A. The change in the relative phase, Δ*φ*(ω) - Δ*φ*(*ω*_0_), for three configurations of the MZ are displayed in Fig. 4 (inset). The group index of the CCW was obtained as


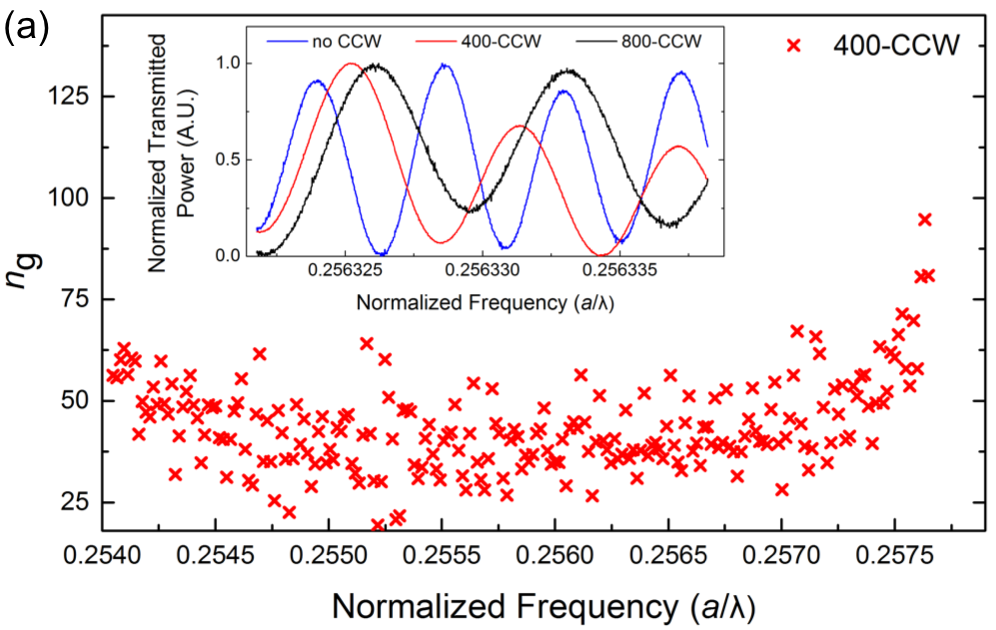


**Figure S3** **|** (a) Calculated *n*_g_ for 400-CCW. Inset: Normalized transmitted power for *λ* ~ 1560.54 nm when in arm-A there was no CCW (blue), 400-CCW (red), and 800-CCW (black).

$$n_{g}= \frac{c}{L_{c}}(\frac{{d\varphi}_{r0}}{d\omega}-\frac{{d\varphi}_{r}}{d\omega}-\frac{n_{gw}L_{w}}{c})$$

with *dφ*_r0_/*dω* and *dφ*_r_/*dω* measured by the MZ fringes. Because the error of the CCW group index (δ*n*_gc_) is related to the error in the measurements as δ*n*_gc_ = (*c/L*_c_) δ[*dφ*_r0_ /*dω - dφ*_r_ /*dω*] and is proportional to 1/*L*_c_, the interferometry method turned out to be better suited than the FSI to measure longer CCWs.

**References**

1. [] McNab, S. J., Moll, N. & Vlasov, Y. A. Ultra-low loss photonic integrated circuit with membrane-type photonic crystal waveguides. *Optics Express* **11**, 2927–2939 (2003). [↑](#endnote-ref-1)
2. [] Thomas, N. L., Houdré, R., Kotlyar, M. V., O'brien, D. & Krauss, T. F. Exploring light propagating in photonic crystals with Fourier optics. *Journal of the Optical Society of America B* **24**, 2964 (2007). [↑](#endnote-ref-2)
3. [] Thomas, N. L. et al. Light transport regimes in slow light photonic crystal waveguides. *Physical Review B* **80**, (2009). [↑](#endnote-ref-3)
